# Psychosocial Motivators for Moderate Drinking among Young Asian Flushers in Singapore

**DOI:** 10.3390/ijerph16111897

**Published:** 2019-05-29

**Authors:** Hye Kyung Kim, Rachel Lim Si En, Dorothy Wong Kang Min

**Affiliations:** Wee Kim Wee School of Communication and Information, Nanyang Technological University, 31 Nanyang Link, Singapore 637718, Singapore; RLIM019@e.ntu.edu.sg (R.L.S.E.); DORO0008@e.ntu.edu.sg (D.W.K.M.)

**Keywords:** Asian flush, alcohol, evidence-based health promotion, theory of planned behaviour

## Abstract

Asians are more susceptible to alcohol flush syndrome and its associated health risks because they are genetically predisposed towards it. Guided by the theory of planned behaviour, this research examined the psychosocial factors associated with moderate alcohol consumption, in order to inform the development of a health campaign targeting young Asian “flushers” in Singapore. We employed a mixed-method design comprising an online survey and focus group discussions. The survey results identified perceived behavioural control as the most salient belief associated with moderate drinking intentions, particularly for Asian flushers. Although Asian flushers had more positive attitudes towards, and perceived behavioural control about drinking in moderation, they were more likely to consider that their peers disapprove of such a practice, compared to non-flushers. Additionally, Asian flushers did not consider themselves as having a higher risk of long-term health effects from alcohol consumption than non-Flushers despite their actual high-risk status. Focus group findings suggest that young Asian flushers have poor knowledge of, and skills associated with moderate drinking, in addition to feeling self-imposed social pressure. The study findings provide practical insights into bridging the information gap on Asian flush and promoting Asian flushers’ drinking in moderation.

## 1. Introduction

Alcohol consumption contributes to global mortality and an increased incidence of short- and long-term health consequences, some of which include the development of seven types of cancer: mouth, pharynx, larynx, oesophageal, breast, liver and colon [1]. Studies have shown that drinkers of East-Asian ethnicity are particularly susceptible to these health risks, because of their genetic predisposition to alcohol flush syndrome [2]. The syndrome is caused by an enzyme deficiency in the liver and is more commonly known as ‘Asian flush’ in the region, where it affects one in three Singaporeans. However, notwithstanding the magnitude of the health impact and the sizable population at-risk in Singapore, there is a lack of research into Asian flushers’ perceptions of, and behaviours concerning alcohol consumption. This makes it difficult to intervene effectively in their alcohol consumption in order to reduce Asian flush-associated health risks. 

For these reasons, this research aims to understand the issue of Asian flush in Singapore better, particularly as it occurs among university students who tend towards excessive alcohol consumption, either as a symbolic entry point into adulthood, or to gain a broad range of life experiences [3]. Previous researchers have emphasised the importance of using a theory when developing health interventions as it helps to identify which specific beliefs to target in order to promote, prevent, or maintain certain health behaviours effectively [4,5]. Therefore, guided by the Theory of planned behaviour [6], this study identified several important psychosocial motivators that could be addressed in health campaigns targeting young Asian flushers in Singapore. We compared flushers and non-flushers in order to understand where the similar and dissimilar characteristics lay, in order to generate better messaging within a campaign. The study’s findings are expected to inform future interventions that address Asian flushers as their priority target audiences.

### 1.1. Asian Flush Syndrome 

Asian flush syndrome refers to an alcohol flush reaction commonly found among Asians, which is caused by an inherited deficiency in the enzyme, aldehyde dehydrogenase-2 (ALDH2). This enzyme is involved in the body’s metabolism of ethanol [7]. A less efficient breakdown of acetaldehyde leads to its accumulation, leading to facial redness, increased heart palpitations, headaches, nausea, and fatigue [8]. After consuming identical amounts of alcohol, Asians with an ALDH2 deficiency (i.e., ‘flushers’) show a blood acetaldehyde concentration five to eighteen times higher than that of non-Asian flushers [9]. This puts Asian flushers at a higher risk of both short- and long-term alcohol-related health problems, ranging from hangovers to the development of cancers and other diseases [7,8,9,10,11,12,13]. As an example, in a study of Asian flushers in Japan and Taiwan, comprising both heavy and light drinkers, were more susceptible to oesophageal cancer than non-Asian flushers [9]. 

Despite Asian flushers’ higher risk standing, most local campaigns on alcohol consumption were not tailored to the needs of flushers in Singapore. For example, a local campaign, entitled “Don’t Choose Binge”, did not segment the population of young drinkers, and prescribed keeping to 4–5 drinks as the recommended behavior—an amount that still poses a significant risk to flushers. Prior research suggests the importance of abstinence for Asian flushers, in order to avoid the health risks that accompany this genetic deficiency. In a study, non-drinkers, who had the ALDH2 deficiency, did not face heightened health risks when they refrained from constant alcohol consumption [14]. Nevertheless, health professionals acknowledge the difficulty young adults have in giving up alcohol completely, so have proposed drinking in moderation, if absolutely necessary. The Health Promotion Board (HPB) [15] in Singapore recommend drinking in moderation, which they define as one standard alcoholic drink for women and two for men, in any single drinking session (no specific time period indicated). This is advised in order to limit aldehyde production and its consequent oversaturation of the liver when metabolising alcohol. This study followed the HPB’s drinking guidelines while examining the psychosocial motivators of moderate drinking among young Asian flushers.

### 1.2. Theoretical Framework: Theory of Planned Behaviour

Ajzen’s theory of planned behaviour (TPB) [6] served as the theoretical framework for this research. Although the TPB has been previously applied to college drinking, and in particularly high-risk drinking [16,17,18], it has not been used to describe young adults’ adherence to a drinking guideline, particularly among those who have Asian flush syndrome. This framework postulates that the enactment of a specific behaviour is dependent on the strength of an individual’s intention to perform it, which is further decided by one’s attitudes, subjective norms, and perceived behavioural control with respect to the behaviour [19]. 

Attitudes, which refers to an overall evaluation of the target behaviour, are formed on the based of underlying beliefs about the positive or negative outcomes of performing the behaviour [6]. Attitudes are typically built upon past experiences [20]; for example, the negative outcomes of excessive drinking, such as hangovers and the positive outcomes which include having a good time with peers. Thus, it is important for interventions to debunk any expectations of the outcome, thus weakening any associations with respect to the benefits, while strengthening associations with the costs of high-risk drinking [21]. Earlier research has found attitudes to be among the strongest predictors of one’s intention to engage in high-risk drinking [22,23]. Thus, the more positive attitude an individual has towards drinking in moderation, the more likely this person is to have a positive intention to engage in moderate drinking.

A “subjective norm” refers to an individual’s perception of others’ expectations of that person’s performing a particular behaviour [6]. Subjective norms are an important concept in college drinking, because college drinking problems arise from an institutionalised campus culture that encourages drinking as a social activity [24]. For young adults, drinking is not solely limited to personal experimentation but also reflects a social desire to fit in [25,26]. Earlier research has found that subjective norms are some of the strongest predictors of high-risk drinking intentions [27], especially where approval from friends rather than family was concerned [20,21]. Thus, the more supportive subjective norms an individual has concerning moderate drinking, the more likely (it is) that this person will have positive intentions to drink in moderation.

Perceived behavioural control (PBC) refers to the perceived ease or difficulty of performing a particular behaviour [6], which stems from beliefs about the presence of obstacles, or facilitating factors for this behavioural performance [5]. According to the TPB, greater PBC linked to behaviour leads to a higher intention to perform that behaviour. Studies have found that a greater perceived capability to refuse drinking leads to lower intentions to engage in high-risk drinking [22] as well as a lowered quantity of drinking, as well as frequency and experience of drinking-related problems [16]. Thus, the greater PBC an individual has over their drinking in moderation, the more likely it is that this person will have a positive intention to drink in moderation.

In addition to these determinants, theoreticians of the TPB postulate risk perceptions, which comprise the perceived susceptibility and severity of risk as distal predictors of intentions, and subsequently behavioural enactment [5]. Perceived susceptibility and risk severity are defined as the probability of experiencing a health risk and its respective seriousness or harmfulness. When an individual considers him/herself not susceptible to health risks and/or when the consequences are not perceived as serious, that individual will not have the intention to drink in moderation. The public’s limited knowledge of Asian flush and its long-term health consequences makes this last component particularly relevant because it is likely to result in low levels of risk perception in the target audience. We investigate Asian flushers’ perceived risk of both the short- and long-term health effects of alcohol consumption and the implications on their forming intentions to drink in moderation. 

### 1.3. Study Overview and Objectives

This research aimed to elucidate the motivations behind an adherence to moderate drinking in order to inform the development of effective campaign strategies that target young Asian flushers in Singapore. To this end, we employed a mixed method of research using both quantitative (survey) and qualitative (focus group) approaches. The institutional review board (IRB) at the hosting university approved both the survey and focus groups for this research.

We used a survey approach, to understand more about young Singaporeans’ current drinking behaviours, risk perceptions and knowledge, and to identify any salient beliefs that could be addressed in campaign messages. Earlier research has puts flushers at a higher risk of the short- and long-term health effects of alcohol consumption [7,8,9,10,11,12,13]. For this reason, we identified Asian flushers and compared their different drinking behaviours, risk perceptions and TPB constructs when compared to non-flushers. The aim of this was to be able to develop campaign messages tailored to the needs of Asian flushers. Focus group research was used to gain a deeper insight into the survey’s results and to examine the efficiency and acceptability of potential campaign strategies, which were based on the survey’s findings. Specifically, we address the following overarching research questions:

RQ1: What are important psychosocial predictors of the intention to drink in moderation? 

RQ2: How do Asian flushers (vs. non-flushers) differently perceive their risks and TPB constructs to engage in moderate drinking?

## 2. Survey Method

### 2.1. Sample and Procedure

We conducted an online survey with a random sample of undergraduate students who were registered at a large public university in Singapore. We randomly selected a list of student email addresses from the entire pool of undergraduate students’ email addresses. An e-mail invitation was sent to each sampled e-mail address with a link to a survey questionnaire on the web. As a study incentive, each student stood a chance to win a lucky draw for $10 shopping voucher.

Students who had consumed alcohol in the past one year and who were above eighteen years of age were eligible to take part in the survey. Out of the 345 individuals who accessed the survey site (a 23% response rate), 208 participants completed the survey. The sample comprised 45.2% male students, and the mean age was 22 (*SD* = 1.92). The majority of respondents (92.8%) were occasional drinkers who drank fewer than four times a month. On average, respondents typically consumed 2.52 drinks (*SD* = 1.88) in one sitting over the past year and their highest consumption on any one occasion was 5.49 drinks (*SD* = 4.91). Self-identified flushers, i.e., those whose faces flushed immediately after drinking one standard alcoholic drink, comprised 56.7% of the respondents. The gender ratio did not differ between flushers and non-flushers (Chi-square = 2.24, *p* = 0.16). 

### 2.2. Measures

We assessed the TPB constructs (attitude, subjective norms, perceived behavioural control, intention), risk perceptions, knowledge about the Alcohol flush reaction, and alcoholic beverage consumption patterns. Unless noted otherwise, all measures were measured using seven-point Likert scales (1 = strongly disagree; 7 = strongly agree). Table 1 presents the correlation between study variables. 

To assess intention, participants reported the extent to which they intended to follow and were likely to exceed (reverse coded) the HPB’s drinking guidelines (*r* = 0.44, *p* < 0.001, *M* = 4.56, *SD* = 1.57). We measured attitudes toward following the HPB’s guidelines on a seven-point scale, using five semantic differential scales (e.g., bad-good, unpleasant-pleasant; alpha = 0.87, *M* = 5.28, *SD* = 1.13). To measure subjective norm, participants reported the extent to which their peers believed flushing was merely a cosmetic issue, and whether they encouraged drinking, and continued drinking even after they flushed (three item alpha = 0.56, *M* = 3.59, *SD* = 1.08). To assess perceived behavioural control, participants reported how easy or difficult it was to follow the guidelines and rated their confidence in following it under different circumstances, such as stress (three item alpha = 0.73, *M* = 4.96, *SD* = 1.56). 

For risk perception, we assessed the respondents’ perceived susceptibility and severity of health consequences of high-risk drinking both in the short-term and long-term. To calculate risk perceptions, we multiplied susceptibility and severity, separately for the short- (*M* = 20.28, *SD* = 11.49) and long-term (*M* = 27.81, *SD* = 11.71) health impacts. To gauge knowledge, we asked to what extent the respondents understood the symptoms of the alcohol flush reaction (*M* = 4.00, *SD* = 1.76), the scientific explanation behind it (*M* = 3.60, *SD* = 1.85), and to what extent it was associated with long-term health risks (*M* = 4.11, *SD* = 1.68). 

## 3. Results

### 3.1. Predicting Intentions to Follow the HPB’s Drinking Guideline

Addressing RQ1, we used ordinary least squares (OLS) regressions to predict the intention to follow the HPB’s drinking (DV). We entered age, gender, status as a flusher, and risk perceptions in the first step, TPB constructs in the second step, and the interactions between Flusher status and TPB constructs in the third step (Table 2). Step 1 explained 13% of the variance in respondents’ intentions to follow the HPB’s drinking guideline. Flushers (vs. non-flushers) had greater intentions to follow the guidelines (*β* = 0.19, *p* = 0.005). Whereas a perceived risk of long-term health effects was a significant predictor of intention (*β* = 0.31, *p <* 0.001), a perceived risk of short-term health effects was *not* associated with intention (to follow the guideline). 

The TPB constructs, entered at Step 2, explained an additional 34% of the variance in intentions. Perceived behavioural control was the strongest predictor of intention (*β* = 0.41, *p* < 0.001), followed by attitude (*β* = 0.27, *p* < 0.001) and then subjective norms (*β* = 0.10, *p* = 0.05). At Step 3, we found significant interaction between perceived behavioural control and Asian flush (*β* = 0.15, *p* = 0.03); compared to non-flushers: the effect of perceived behavioural control on intention was stronger among flushers.

### 3.2. Comparisons by Asian Flush

Using a series of one-way analysis of variance (ANOVA), we further compared flushers and non-flushers’ risk perceptions, TPB constructs and drinking behaviours addressing RQ2 (Table 3). Although risk perception did not differ between the two groups, flushers consumed significantly less alcohol than non-flushers in regards to both the typical (*M _flusher_* = 2.17, *M _non-flusher_* = 2.99, *p* = 0.001) and the highest (*M _flusher_* = 4.54, *M _non-flusher_* = 6.73, *p* = 0.001) amounts they consuming in one sitting over the past year. However, both the typical and the highest amounts that flushers consumed exceeded the HPB’s drinking guideline.

Flushers had a more positive attitude (*M _flusher_* = 5.43, *M _non-flusher_* = 5.09, *p* = 0.03) and perceived behavioural control (*M _flusher_* = 5.34, *M _non-flusher_* = 4.46, *p* < 0.001) compared to non-flushers. On the contrary, flushers had less supportive subjective norms than non-flushers (*M _flusher_* = 3.46, *M _non-flusher_* = 3.75, *p* = 0.05). 

### 3.3. Knowledge of Asian Flush

Most of the respondents (87.3%) considered five or more drinks as the threshold of excessive drinking. Our results showed a general poor knowledge about Asian flush, with 60% of the respondents reporting that they do not know about the scientific explanation behind the redness that occurred. Around half of the respondents (51.9%) reported that they were unable to identify the symptoms of the alcohol flush reaction. Flushers were more confident in identifying the symptoms of the alcohol flush reaction than non-flushers (*M _flusher_* = 4.32, *M _non-flusher_* = 3.57, *p* = 0.002). Only 42% of the respondents believed there was a link between the alcohol flush reaction and long-term health effects.

## 4. Focus Group

### 4.1. Method

We conducted two focus group discussions to gather insights that could explain key findings from the survey and to test potential message appeals and campaign strategies. Focus groups are meant to identify underlying beliefs and reasons for a behaviour, rather than to arrive at a consensus. Using a snowball sampling, eleven undergraduate students, all ethnic Chinese aged between 23 to 26 years, were recruited (session 1, *n* = 6; session 2, *n* = 5) for each hour-long discussion. The focus group included an equal mix of flushers and non-flushers and of females and males. Among the flushers were those who abided by or exceeded the HPB’s guidelines in their drinking sessions. 

Participants were informed that it was not necessary to reach a consensus and it was okay to have different opinions. The moderator started with broad, pre-determined questions (informed by the survey’s findings), that mostly offered all participants equal opportunities to speak up. Focus groups were audio-recorded and then fully transcribed for the analysis. The researchers conducted a thematic analysis [28] to identify salient patterns and themes, which were then classified into different topical categories. All FG participants received a $10 shopping voucher for their study participation.

### 4.2. Results

#### 4.2.1. Adherence to Drinking Guidelines 

The focus group discussions found that the respondents, both flushers and non-flushers, demonstrated poor skills in keeping to drinking limits such as the HPB’s guidelines. Generally, the respondents lacked an accurate understanding of what constituted a standard alcoholic drink, which led to the overconsumption of beverages with a higher alcohol content, such as vodka and soju. Objective measures were rarely used to quantify excessive drinking; instead, many used subjective measures of drunkenness to determine whether they were drinking out of moderation. These judgments were mainly based on physiological reactions, such as an increased rate of heart palpitations, and the clarity of their mental or emotional state, such as the sense of ‘losing control’. Some of the comments elicited from peers were:

“I think drinking in moderation means as long as I can get back home myself.”

“It also depends on what people tell you. So, for example, if they say you look flushed, then they will suggest that maybe you should stop.”

#### 4.2.2. Social Pressure 

There was a general consensus that respondents did not explicitly coerce their drinking peers into consuming more alcohol than they wanted to. Nevertheless, unspoken pressures remained in typical drinking sessions. Most tended to overestimate the social pressure and perceived that their peers expected them to drink more than what was actually expected, resulting in a self-imposed pressure to drink:

“If everybody’s getting hammered and they’re laughing, but you are like ‘what’s going on’. If you don’t drink, it’s just not fun. So, you should drink”.

These self-imposed pressures are especially pronounced when meeting new people or being offered drinks by seniors. Respondents admitted to drinking out of routine to be accepted by social groups. These same instances were also the occasions where they exceeded drinking limits; be they HPB’s guidelines, or those set for themselves.

“I think I used to feel the pressure also, and more when I meet new people. When I meet new people, I feel like there’s something to show.”

“When the seniors give you the drink, it’s like you’re obliged to finish it, out of respect more than anything.”

#### 4.2.3. Message Appeal 

Participants, particularly Asian flushers, mentioned that, given the constant bombardment of information with respect to health issues, they were desensitised and averse to the use of fear appeals.

“I agree with not using fear messaging. We are too used to being scared. They (HPB) have been trying to scare us all our lives, so I don’t think it is going to work.”

Instead, respondents were found to be more receptive to the use of objective measures in understanding drinking in moderation. Notably, flushers appreciated the use of factual data, in the form of infographics that could help them visualise the cost-benefit analysis of reducing their alcohol intake, thereby motivating a positive behavioural change.

“We are desensitised. I was thinking of something more logical as well, something like: we are exposed to how many kinds of cancers, and then this small, little drink can increase your cancer risks by that much more.”

“Perhaps you could say, ‘If you’re prone to Asian flushing, drink x beers or y shots of hard liquor’. That extra infographic or a recommended amount of intake would help”.

#### 4.2.4. Peer Support 

Non-flushers generally considered themselves capable of promoting HPB’s guidelines among their Flusher friends. However, the fear of being labelled a buzzkill translated to a general reluctance to discuss the health consequences of drinking for flushers, especially during the drinking session itself. This was compounded by a perception of the long-term health risks as a distant concept that could not be resolved with immediacy.

“It’s quite a buzzkill when you say: ‘Did you know that you can contract oesophageal cancer?’ while people drink.”

“If you tell someone, ‘You have a high risk of cancer’, it doesn’t really do much, it’s not like he can do anything about it there and then.”

Still, non-flushers reported that they would be willing to promote moderate drinking to flushers, outside of the drinking environment, if it affected flushers who were close to them.

“I would tell my best friend who flushes easily and drinks a lot. But in a social setting, I wouldn’t want to say, ‘Hey, you’re going to get cancer’. At least not at that time, but maybe on another occasion, at a better time.”

## 5. Discussion

This research aimed to provide useful information for the development of a health campaign, addressing the issue of Asian flush among university students in Singapore. To this end, guided by the TPB, we examined the psychosocial factors associated with adherence to the HPB’s drinking guidelines, in order to identify important beliefs that might be targeted in campaign messages. In particular, this study compared risk perceptions and psychosocial factors, between flushers and non-flushers, to offer insights into message tailoring. To our knowledge, this is the first study to examine Asian flushers’ drinking behaviours and their motivations for moderate drinking. Based on a mixed method approach, as recommended in other health research [29,30], our findings offered practical insights into the design of effective campaigns that target Asian flushers in Singapore, and more broadly, in Asian and multi-ethnic countries.

### 5.1. Risk Perceptions and Knowledge on Asian Flush

The majority of the respondents (93%) were occasional drinkers, typically consuming 2.52 standard drinks in one sitting. However, 87% of the respondents considered having five or more drinks excessive drinking, which is a much higher number than the HPB’s guidelines (one drink for women and two drinks for men). It also exceeds the defined threshold for high-risk drinking (four drinks for women and five drinks for men) [31]. Furthermore, the focus group findings suggested that respondents did not use objective thresholds to determine whether they were drunk or not. Generally, they made their decisions about when to stop drinking, based on their physiological reactions, mental state and peer’s comments. More importantly, more than half of the respondent (58%) did not believe there was a link between alcohol flush reaction and its long-term health effects. 

Interestingly, flushers tended to consume less alcohol than non-flushers, potentially because of the alcohol flush reactions they had experienced [32]. Nevertheless, flushers did not consider themselves as having a higher risk of experiencing alcohol-induced short- and long-term health effects, as compared to non-flushers—despite their actually having a higher risk status. At the same time, the perceived risk of the long-term effects of alcohol consumption was positively associated with intentions to follow the HPB’s guideline, but not the perceived risk of short-term effects. These results highlight the importance of addressing the information gap for Asian flushers. Awareness needs to be raised concerning their higher risk of suffering long-term alcohol-related health effects and an objective threshold for excessive drinking is needed, which is tailored to flushers, in order to lower their risk. 

To enhance flushers’ risk perceptions, campaign messages could include the scientific explanation behind Asian Flush, especially given that 60% of the respondents were not aware of it. Because Asian flushers are at a higher risk for various types of cancer [7,9,11], guidelines including information about the genetic risk and a recommendation for genetic assessment (i.e., genotyping ALDH2) might be beneficial [33]. Additionally, nearly half of the respondents were unsure about the symptoms of Asian flush reaction, although flushers were more knowledgeable than non-flushers. Research suggests that young adults are more likely than older adults to experience psychological reactance, because of their need to maintain autonomy [34]. Our focus group findings also suggest that university students are generally averse to the idea of an appeal to fear. Thus, when developing campaign messages, an informational approach may be more suitable for addressing the issue of Asian Flush than a fear appeal. In terms of delivery, our focus group findings suggest that students prefer not to discuss the health effects of alcohol while they are drinking because they consider it socially inappropriate. If a message is to be delivered during drinking sessions, campaigners should develop strategies that overcome this resistance. Future research should aim to confirm the efficacy of different messages, appeals, and delivery approaches further, using experimental designs. 

### 5.2. Motivational Factors to Moderate Drinking 

In line with the TPB, the results showed that improvements in attitudes, perceived behavioural control, and subjective norms can help to enhance university students’ intentions towards moderate drinking that follows the HPB’s drinking guideline. Perceived behavioural control, in particular, was most strongly associated with an intention to follow the drinking guideline. This suggests that campaign messages that address perceived behavioural control - for example, by instructing students on how to avoid excessive drinking - would be the most effective at improving university students’ intention to drink in moderation. More importantly, this strategy is likely to be more effective for Asian flushers (vs. non-flushers), given the significant interaction that our study identified between perceived behavioural control and Asian flush.

Conversely, flushers generally had a greater intention to follow the drinking guidelines compared to non-flushers. This was explained by flushers’ more positive attitude towards and greater perceived behavioural control about following the guidelines. Flushers were more likely to be favourable towards and experienced at restricting their drinking amount potentially because of past episodes of alcohol flush reaction [32]. However, our findings suggest the importance of building supportive norms for moderate drinking, particularly among Asian flushers. Compared to non-flushers, flushers were more likely to consider that their peers do not support their drinking in moderation. Our focus group findings also suggest that flushers tend to overestimate the social pressure and perceive that their peers expect them to drink more than what is actually expected. 

Previous research into the health risks associated with excessive alcohol intake has also identified social norms as an integral factor in shaping a safer drinking environment, particularly for Asian flushers [35,36,37]. In a recent study, university students reported greater willingness to intervene a flushing drinker when the flusher was a female, a close friend and they were drinking for fun [38]. Along with being a close friend, our focus group further identified the reluctance to intervene a flushing drinker while drinking because of the fear of being labelled a buzzkill. For Singaporeans, who thrive in a collectivistic culture that prioritises the concept of ‘we over me’, a small shift in perceived social pressure would be beneficial in forming positive intentions to engage in moderate drinking. To this end, campaign messages could advocate a collective environment that encourages safer and healthier drinking, asserting that it is socially acceptable for Asian flushers to reject drinks that go beyond their limits.

### 5.3. Study Limitations

This study had several limitations. First, although we employed a random sampling of undergraduate students enrolled in a large university in Singapore, there was a low response rate (23%). Using the enrolment statistics in 2018, the profile of our sample is similar to the university’s student population in terms of gender and age. However, it is difficult to determine whether a nonresponse bias is present. Additionally, because the survey and focus groups were conducted solely in one university, our results might not be an accurate representation of all university students in Singapore. For example, universities that do not have residences for its student population might report different normative beliefs when it comes to drinking. To improve the generalizability of this survey’s findings, future work should improve the response rate, for example by providing higher incentives, and could randomly sample the true population of interest. Future focus group studies investigating university students’ beliefs on drinking could also include more groups and consider broader psychographics, by including participants from different social backgrounds. 

Second, respondents were asked to recall their highest drinking occurrence within the past year and to express it in terms of standard alcoholic drinks. This may not have been a familiar concept to them, even though we provided relevant definitions within the survey. Although we clearly stated that participants’ responses are anonymous and confidential, there’s a potential for social desirability bias in reporting drinking amount and flusher status. Additionally, participants may not have accurately remembered their past alcohol consumption. In particular, impaired judgement caused by the influence of alcohol might have led to an over- or under-reporting of actual quantities of alcohol consumption. Future research should seek to improve the measurement of alcohol consumption, for example, by employing actual behavioural assessment.

## 6. Conclusions

Given the local information gap and the low topical awareness surrounding the issue of Asian flush, this research offers practical insights into the development of effective campaigns that target Asian flushers. Knowledge and the perception of risk with respect to Asian flush were identified as lacking among young adults in Singapore, therefore campaigners should focus on filling the information gaps concerning this topic. Although we focused on young Singaporeans, our findings also offer practical insights for dealing with flushers in other Asian and multi-ethnic countries. Specifically, campaigners should identify Asian flushers within the target population and provide them with tailored-information designed to enhance their efficacy and supportive norms regarding moderate drinking to achieve sustained behavioural changes. Evidence-based communication strategies, culturally-relevant content and innovative initiatives can work to create healthy conversations on the issue of Asian flush.

## Figures and Tables

**Table 1:** Bivariate Correlations of Study Variables.

	1.	2.	3.	4.	5.
1. Long-term risk perception					
2. Short-term risk perception	0.51 ***				
3. Attitude	0.33 ***	0.18 **			
4. Subjective norms	−0.02	−0.13	0.10 ***		
5. Perceived behavioural control	0.27 ***	0.22 ***	0.57 ***	0.10	
6. Intention	0.33 ***	0.21 **	0.57 ***	0.16 *	0.63 ***

* *p* < 0.05. ** *p* < 0.01. *** *p* < 0.001.

**Table 2:** Ordinary Least Squares Regression Predicting Intention.

Predictors	*β*	*SE*	*t*
Block 1			
Age	0.03	0.06	0.38
Female (vs. Male)	0.05	0.23	0.73
Flusher (vs. Non-Flusher)	0.19 **	0.21	02.84
Long-term risk perceptions	0.31 ***	0.09	−1.69
Short-term risk perceptions	0.02	0.01	4.11
Adjusted *R*^2^	0.13 *F*(5, 202) = 7.18		
ANOVA			
Block 2			
Attitude	0.27 ***	0.09	4.30
Subjective norms	0.10 *	0.08	1.94
Perceived behavioural control	0.41 ***	0.07	6.40
Adjusted *R*^2^	0.47 *F*(8, 199) = 23.92		
ANOVA			
Block 3			
Attitude*Flusher	−0.07	0.18	−1.08
Subjective norms*Flusher	−0.04	0.16	−0.79
Perceived behavioural control*Flusher	0.15 *	0.15	2.21
Adjusted *R*^2^	0.48 *F*(11, 196) = 18.11		
ANOVA			

* *p* < 0.05. ** *p* < 0.01. *** *p* < 0.001.

**Table 3:** Risk Perceptions, TPB Constructs and Alcohol Consumption by Asian Flush.

	Asian Flush				*F*
	Flushers (*n* = 118)		Non-flushers (*n* = 90)		
	*M*	*SD*	*M*	*SD*	
**Risk perception**					
Long-term	28.24	12.25	27.24	10.99	0.37
Short-term	21.36	12.39	18.88	10.08	2.39
**TPB constructs**					
Attitude	5.43	1.08	5.09	1.18	4.66 *
Subjective norms	3.46	1.06	3.76	1.09	3.81 *
Perceived behavioural control	5.34	1.22	4.46	1.81	17.72 ***
**Alcohol consumption**					
Typical amount	2.17	1.51	2.99	2.19	10.42 **
Highest amount	4.54	3.60	6.73	6.03	10.62 **

* *p* < 0.05. ** *p* < 0.01. *** *p* < 0.001.
